# Regional Anesthesia Techniques for Shoulder Surgery in High-Risk Pulmonary Patients

**DOI:** 10.3390/jcm12103483

**Published:** 2023-05-16

**Authors:** Bradley H. Lee, William P. Qiao, Stephen McCracken, Michael N. Singleton, Mikhail Goman

**Affiliations:** 1 Department of Anesthesiology, Critical Care & Pain Management, Hospital for Special Surgery, New York, NY 10021, USA; qiaow@hss.edu (W.P.Q.); mccrackens@hss.edu (S.M.); singletonm@hss.edu (M.N.S.); gomanm@hss.edu (M.G.); 2 Department of Anesthesiology, Weill Cornell Medicine, New York, NY 10021, USA

**Keywords:** diaphragm-sparing, phrenic nerve sparing, shoulder surgery, brachial plexus block, COPD

## Abstract

Patients with pre-existing pulmonary conditions are at risk for experiencing perioperative complications and increased morbidity. General anesthesia has historically been used for shoulder surgery, though regional anesthesia techniques are increasingly used to provide anesthesia and improved pain control after surgery. Relative to regional anesthesia, patients who undergo general anesthesia may be more prone to risks of barotrauma, postoperative hypoxemia, and pneumonia. High-risk pulmonary patients, in particular, may be exposed to these risks of general anesthesia. Traditional regional anesthesia techniques for shoulder surgery are associated with high rates of phrenic nerve paralysis which significantly impairs pulmonary function. Newer regional anesthesia techniques have been developed, however, that provide effective analgesia and surgical anesthesia while having much lower rates of phrenic nerve paralysis, thereby preserving pulmonary function.

## 1. Introduction

Patients who present for surgery are often affected by various pulmonary pathologies such as asthma, chronic obstructive pulmonary disease (COPD), obstructive sleep apnea (OSA), interstitial lung disease, and pulmonary hypertension [1]. COPD is a leading cause of morbidity and mortality in many countries with an estimated prevalence near 10.1% [2]. Obstructive sleep apnea is also common and affects up to 24% of the United States (USA) population [1]. Patients with pulmonary disease are prone to risks of hypoxemia, pneumonia, and respiratory failure after surgery [1]. These pulmonary complications result in increased morbidity and long-term mortality, as well as higher medical costs [1].

Surgery for treatment of shoulder conditions ranging from instability to arthritis is common, and the volume of shoulder surgery has increased rapidly in the USA. Since reverse total shoulder arthroplasty was approved by Food Drug Administration (FDA) in 2003, the number of annual shoulder arthroplasties has grown significantly with a 2.5-fold increase between 2000 and 2008 [3]. Shoulder arthroscopy is often performed in ambulatory settings, and the number of procedures increased nearly 4-fold between 1996 and 2006 [4]. During this time, general anesthesia was utilized more often than regional anesthesia, though regional anesthesia was used more frequently over time [4]. 

There are concerns related to general anesthesia in patients with pulmonary disease who undergo surgery. For example, general anesthesia in those with underlying pulmonary pathology may lead to complications of postoperative atelectasis, hypoxia, pneumonia, or postoperative intubation [5]. In addition, there are inherent risks related to general anesthesia such as laryngospasm and bronchospasm in these patients [5]. Patients also experience significant pain after surgery, and high doses of opioids may cause excessive sedation and worsen respiratory depression increasing the risk of hypoxemia in patients with OSA [6].

Regional anesthesia for shoulder surgery involves anesthetizing nerves of the brachial plexus that innervate the shoulder [7]. Techniques such as the interscalene block (ISB) have traditionally been utilized and provide effective analgesia after shoulder surgery [7]. However, a major limitation of the ISB is that the phrenic nerve is anesthetized resulting in phrenic nerve paralysis. Therefore, this technique cannot be tolerated in patients with underlying pulmonary disease as it severely reduces pulmonary function.

Fortunately, there are recent advances in regional anesthesia that allow selective anesthetization of the nerves necessary to provide analgesia for shoulder surgery while preserving diaphragmatic function [7]. Here we review traditional regional anesthesia techniques used for shoulder surgery, as well as novel techniques that may be utilized in high-risk patients who have underlying pulmonary pathology. 

## 2. Shoulder Anatomy and Innervation

The shoulder is formed by two joints—The acromioclavicular joint formed by the acromion process of the scapula and the clavicle, and the glenohumeral joint formed by the glenoid process of the scapula and the humeral head [8]. The only bony connection of the upper extremity to the thorax is the acromioclavicular joint, necessitating a large group of muscles to stabilize the shoulder joint. Muscles contributing to shoulder stability and movement include supraspinatus, infraspinatus, teres major and minor, subscapularis, deltoid, biceps, triceps, pectorals major and minor, latissimus dorsi, and corocobrachialis [8]. This mechanical complexity is accompanied by similarly complicated contributions from the nervous system.

**Figure 1:**
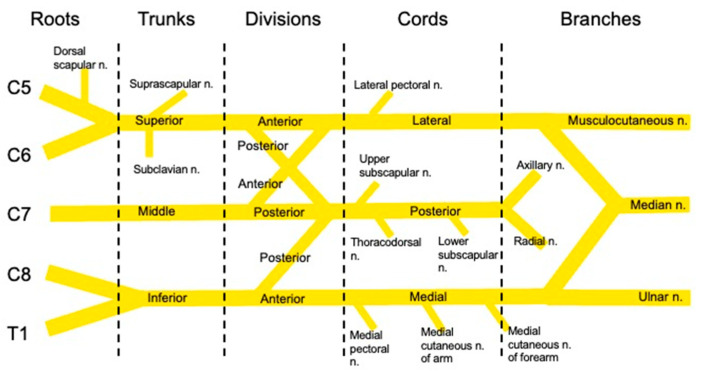
Diagram of brachial plexus. n. = nerve; C5 = Cervical nerve root 5; C6 = Cervical nerve root 6; C7 = Cervical nerve root 7; C8 = Cervical nerve root 8; T1 = Thoracic nerve root 1.

The shoulder joint is innervated by branches of the brachial plexus which include the suprascapular nerve, axillary nerve, nerve to subscapularis, and lateral pectoral nerve (see Figure 1) [9]. The suprascapular nerve arises from the ventral rami of cervical nerve roots (C5 and C6) and is a branch of the superior trunk of the brachial plexus. The suprascapular nerve is a mixed motor and sensory nerve providing motor innervation to the supraspinatus and infraspinatus muscles. The suprascapular nerve provides sensory innervation to the acromioclavicular joint, glenohumeral joint, and ligaments of the shoulder [9,10]. The axillary nerve also originates from the ventral rami of C5 and C6 and is a terminal branch of the brachial plexus. The axillary nerve is a mixed motor and sensory nerve providing motor innervation to the deltoid and sensory contributions to the glenohumeral joint [9]. Other nerves involved in the sensory innervation of the shoulder joint include the nerve to subscapularis and the lateral pectoral nerve both of which arise from ventral rami of C5 and C6 [9]. Cutaneous innervation is also important to consider in performing regional anesthesia. The cutaneous innervation of the shoulder is primarily provided by branches of the brachial plexus with minor contributions from thoracic nerve roots. The supraclavicular nerve arises from cervical 3 and 4 nerve roots and provides cutaneous innervation to the cape of the shoulder [11]. The axillary nerve provides cutaneous innervation over the lateral aspect of the shoulder [11]. The intercostobrachial nerve typically arises from the second intercostal nerve and is responsible for cutaneous innervation of the axilla and medial aspect of the upper arm [11]. 

**Figure 2:**
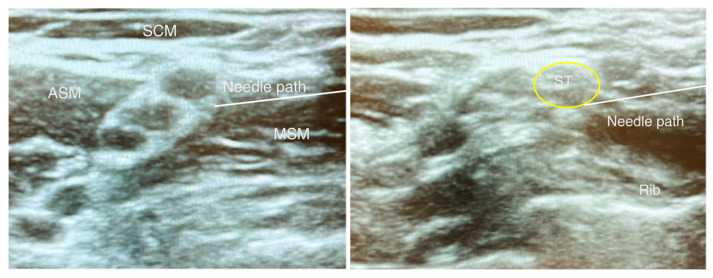
Interscalene and superior trunk blocks. SCM = Sternocleidomastoid muscle, ASM = Anterior scalene muscle, MSM = Middle scalene muscle, ST = Superior trunk. Yellow circle = Superior trunk; White line = Needle path for performing nerve block.

Regional anesthesia for shoulder surgery must, therefore, result in anesthesia of the following: suprascapular nerve, axillary nerve, nerve to subscapularis, lateral pectoral nerve, and supraclavicular nerve [9,11]. The traditional approach to regional anesthesia for shoulder surgery is the interscalene block which anesthetizes C5 and C6 nerve roots by depositing local anesthetic between these nerve roots (see Figure 2). While this block provides effective analgesia, an inevitable consequence is blockade of the ipsilateral phrenic nerve which occurs virtually 100% of the time resulting in hemi-diaphragmatic paralysis (HDP) [12]. The phrenic nerve originates from the ventral ramus of C4 with contributions from C3 and C5. It courses over the anterior scalene muscle and lies near the brachial plexus [12]. Hemi-diaphragmatic paresis is generally well tolerated in healthy patients; however, careful consideration of anesthetic technique is paramount when caring for patients with pre-existing pulmonary disease. Because of this limitation, newer techniques have been developed that may avoid phrenic nerve paresis while providing analgesia for shoulder surgery.

## 3. Novel Diaphragm-Sparing Regional Techniques

### 3.1. Superior Trunk Block

See Table 1 for summary of regional techniques. The superior trunk block (STB), first described in 2014, is performed where C5, C6 nerve roots converge into the superior trunk [13]. Local anesthetic is deposited at the trunk before the suprascapular nerve branches and travels laterally and posteriorly towards the suprascapular notch [13] (see Figure 2). Because the superior trunk is located farther from the phrenic nerve, the risk of HDP is potentially decreased. At the level of the cricoid cartilage, the phrenic nerve is within 2 mm of the brachial plexus, and the distance between the two structures increases as the brachial plexus moves distally down the neck [13]. 

Kim and colleagues compared STB to ISB in a randomized trial of 126 patients undergoing arthroscopic surgery. Patients with STB reported noninferior maximal pain scores in the recovery room, as well as utilizing similar opioid amounts and lower pain scores on postoperative day 1. Importantly, the authors found the STB group had significantly lower risk of complete HDP (4.8% vs. 71.4% in the ISB group) [7]. 

A more recent study by Robles et al., specifically studied the effect of STB on diaphragm function in shoulder surgery. Out of 30 patients receiving STB for arthroscopic shoulder surgery, 33% of patients developed complete HDP with an additional 26.7% of patients developing partial HDP [14]. Among patients with affected diaphragm function, none had significantly altered oxygen saturation; however, 38.9% reported dyspnea and 83.3% had reduced breath sounds on exam [14]. While STB provides similar analgesia with less HDP compared with ISB, there is still possibility of diaphragmatic dysfunction which is important to consider in patients with compromised respiratory status. 

There is significant difference in the incidence of HDP that may be associated with STB. For example, the lowest incidence of HDP with STB is reported to be 4.8% while it has also been reported to be as high as 76.3% [7,15]. The volume of local anesthetic appears to be an important factor as larger volumes of local anesthetic are associated with greater likelihood of HDP [16]. In comparing two studies by Kim et al., and Kang et al., similar volumes (15 mL) and concentrations (0.5%) of local anesthetics were used, though incidence of HDP was quite different (4.8% vs. 76.3%) [7,15]. The discrepancy in findings despite similar concentrations and volumes of local anesthetic seem to suggest that other factors may be implicated. For example, the rate of injection is not measured, but faster injection would theoretically result in greater proximal spread and risk of HDP. Related to this, intermittent injection versus continuous injection could potentially result in different local anesthetic spread if intermittent injection is performed as bolus dosing. Lastly, both studies performed injection proximal to the take-off of the suprascapular nerve; however, slight differences in the level of injection and proximity to the phrenic nerve may contribute to risk of HDP. 

### 3.2. Combined Axillary and Suprascapular Nerve Block

Combined axillary and suprascapular nerve block (SSNB-ANB) was first described in 2007 as an alternative to ISB for shoulder surgery [17]. Of the nerves innervating the shoulder, axillary and suprascapular nerves make up the majority of the innervation. Selective blockade can be performed at the posterior humerus for the axillary nerve, and the suprascapular fossa for the suprascapular nerve [17]. These nerves can also be approached via anterior approach with the suprascapular nerve identified lateral to the brachial plexus below the omohyoid muscle and the axillary nerve identified in the axillary fossa [18,19].

Two randomized controlled trials (RCTs) assess the efficacy of SSNB-ANB compared with ISB in arthroscopic shoulder surgery. Dhir et al., reported that 60 patients undergoing arthroscopic shoulder surgery had worse pain control in the immediate postoperative period with SSNB-ANB compared with ISB; however, SSNB-ANB provided better pain control at 24 h [20]. Opioid consumption was also higher in the SSNB-ANB group. Neuts et al., had similar results reporting inferior pain control and increased opioid consumption in the SSNB-ANB group in the immediate postoperative period compared to ISB [21]. 

A significant limitation of SSNB-ANB is its incomplete analgesic coverage as it ignores contributions of the lateral pectoral, subscapular, and musculocutaneous nerves [22]. In both randomized controlled trials discussed above, patients received SSNB-ANB in addition to general anesthesia. Thus, the evidence supporting SSNB-ANB for surgical anesthesia is lacking. Patients undergoing shoulder surgery utilizing SSNB-ANB may still require general anesthesia and be exposed to certain risks inherent with general anesthesia. 

### 3.3. Combined Infraclavicular and Suprascapular Nerve Block

Combined infraclavicular and suprascapular nerve block (ICB-SSNB) is a technique that may address inadequacies of the SSNB-ANB approach in providing analgesia. Infraclavicular block (ICB) targets the brachial plexus at the level of the cords from which the lateral pectoral, subscapular, and axillary nerve derive [23,24]. These nerves (with exception of the axillary nerve) are missed by the SSNB-ANB approach described previously. ICB-SSNB blocks the major branches that innervate the shoulder with a low risk of HDP. Spread of local anesthetic using fluoroscopy after ICB demonstrates local anesthetic spread confined to below the clavicle [25].

In patients undergoing arthroscopic shoulder surgery, ICB-SSNB resulted in higher pain scores in the immediate 30 min postoperative period and small but significant higher morphine consumption at 24 h compared with ISB; however, none of the patients suffered HDP after ICB-SSNB while ISB resulted in 90% rate of HDP [26]. Similar results were found by Pavoni et al., and ICB-SSNB group achieved full surgical anesthesia without requiring general anesthesia for arthroscopic shoulder surgery and 0% HDP compared to the ISB group [27].

Despite encouraging evidence of ICB-SSB’s diaphragmatic sparing attributes, evidence of HDP in setting of ICB does exist. Some studies have reported 3–5% rate of HDP [28]. While ICB vastly reduced the risk of HDP, current evidence does not support the complete avoidance of this small risk [29]. 

### 3.4. Costoclavicular 

Costoclavicular nerve block (CCB) is another alternative approach to ICB. It is performed in the costoclavicular space where the three cords of the brachial plexus are clustered together, providing a consistent and reliable approach for blockade by local anesthetics [30].

Several RCTs have compared CCB to ISB. Aliste et al., compared CCB with ISB for arthroscopic shoulder surgery and reported similar pain scores within 24 h [31]. However, other RCTs have reported inferiority of time to full motor block onset and immediate pain control postoperatively when compared with ISB or STB for arthroscopic shoulder surgery [32,33].

In terms of diaphragmatic effects, Aliste et al., reported 0% HDP in CCB compared with 100% in the ISB group [31]. A larger RCT published recently with over 200 patients showed CCB produced comparable pain scores versus ISB, though CCB group had a 7.6% rate of HDP compared with 92.5% in the ISB group [32]. Patients in this study did not require general anesthesia suggesting a potential advantage of CCB in high-risk pulmonary patients who might benefit from avoiding general anesthesia. 

## 4. Postoperative Complications in Patients with Pulmonary Disease

There are several factors to consider for patients with pulmonary disease who undergo shoulder surgery. These patients may be especially prone to risks associated with anesthesia. Those with COPD have decreased pulmonary reserve and are susceptible to difficulties such as requiring postoperative ventilation. It is often challenging to wean these patients from mechanical ventilation, and they may need more aggressive pulmonary support postoperatively using continuous positive airway pressure (CPAP) or high flow oxygen and may even require reintubation. In a retrospective review by Hausman et al., it was shown that patients with COPD who underwent general anesthesia compared with regional anesthesia had a higher incidence of postoperative pneumonia, prolonged ventilator dependence, and unplanned postoperative intubation [34]. In another retrospective review examining patients undergoing total hip arthroplasty, patients with COPD had longer lengths of stay and higher likelihood to be discharged to a subacute care facility, as well as higher risks of pneumonia, septic shock, unplanned intubation, and 30-day readmission [35]. 

Obstructive sleep apnea (OSA) is also common and often undiagnosed [36] Patients with OSA have increased risks of pneumonia, postoperative apnea, and thromboembolic complications [37]. They may have impaired immunity and neutrophil function which predisposes them to pneumonia after surgery. In addition, increased fibrinogen levels and blood viscosity raises the risk for deep vein thrombosis (DVT) and pulmonary embolus (PE). A retrospective review identified patients undergoing total knee and total hip replacements with OSA to have greater 90-day readmission rates. Patients with OSA who undergo total shoulder arthroplasty may be at higher risk for experiencing postoperative dyspnea, DVT and PE, infection, and paralytic ileus [38]. 

## 5. General Anesthesia versus Regional Anesthesia

Historically, most shoulder surgery was performed under general anesthesia [39]. However, after Dr. Winnie described the modern approach to the interscalene block, a plethora of anesthetic and analgesic options have been developed [40,41,42,43]. This allows anesthesiologists to choose between general anesthesia, combined general and regional anesthesia, or primary regional anesthesia based on patient characteristics, surgeon preferences, institutional constraints, and proceduralist technical expertise. Having a variety of options to manage patients presenting for shoulder surgery, particularly those with complicated lung physiology, allows the anesthesiologist to provide optimal care for their patients in a variety of circumstances.

Studies comparing the various techniques are numerous. Most point to a reduction in anesthesia recovery time, increased operating room efficiency, less post-operative nausea and vomiting, and reduced opioid consumption when regional blocks are utilized for ambulatory shoulder surgery [42,44,45,46,47]. Intraoperatively, by avoiding the positive pressure ventilation typically associated with general anesthesia, primary regional anesthetics allow for increased venous return and a reduction in cerebral hypoxic events that are of particular concern in the beach chair position [48,49,50].

However, in patients with respiratory pathology who present for shoulder surgery, decisions regarding type of anesthesia become more complex. The effects of general anesthesia on the respiratory system are well described [51]. Induction of general anesthesia and the administration of non-depolarizing neuromuscular blockade both lead to reductions in functional residual capacity and worsening of ventilation-perfusion matching. Residual anesthetics and neuromuscular blockade also contribute to pharyngeal and accessory muscle weakness leading to hypoventilation and obstruction. This in combination with absorption atelectasis resulting from high inspired oxygen concentrations leave many patients with impaired respiratory physiology post-operatively [51,52]. In addition, placing an airway for general anesthesia can cause tracheal or laryngeal irritation leading to bronchospasm or laryngospasm in individuals with reactive airways.

Regional blocks for shoulder surgery are not without negative effects on respiratory physiology. It has been known for some time that the interscalene block results in phrenic nerve blockade leading to ipsilateral diaphragmatic paresis [53]. Phrenic nerve paresis may not be completely avoidable even by reducing the volume, reducing the concentration, or by blocking more distally along the brachial plexus with novel techniques [54,55,56]. Even the infraclavicular block comes with a non-zero, albeit much lower, risk of phrenic nerve block [29]. Phrenic nerve blockade effects are seen on force vital capacity (FVC) and forced expiratory volume in one second (FEV1) with reductions of these parameters in the 20–30% range, which is consistent with studies on respiratory physiology after surgical resection of the phrenic nerve [56,57,58]. It is also important to note a difference in the degree of phrenic nerve blockade. Many regional blocks result in partial phrenic nerve paralysis, where diaphragmatic excursion is reduced 25–75% from baseline. This contrasts with complete paralysis, where diaphragmatic excursion is reduced greater than 75% from baseline, or even exhibits paradoxical motion. While the relationship between degree of phrenic nerve paralysis and its effects on respiratory symptoms and pulmonary function parameters is not well established, logically a partial paralysis would be better tolerated.

Despite the limitations of regional anesthetics in pulmonary disease, clinicians have adapted their techniques to minimize effects on respiratory physiology. While reducing volume and concentration and choosing more distal blocks do not eliminate the risk of negative effects on pulmonary physiology, the lower risk of phrenic nerve blockade they provide often makes them part of an optimal anesthetic strategy for high-risk pulmonary patients. 

## 6. Conclusions

The decision to utilize general anesthesia, regional anesthesia, or both depends on patient comorbidities, surgical needs, and the anesthesiologist’s comfort level with the array of regional anesthesia techniques. In most instances, even if a patient appears unlikely to tolerate a complete unilateral phrenic nerve paralysis from an interscalene block, an anesthetic involving regional techniques can be designed that spares the phrenic nerve and achieves the anesthetic or analgesic goals of the anesthesiologist and patient.

In addition to the regional anesthetic technique, the volume of local anesthetic used is an important factor that may influence the risk of potential hemi-diaphragmatic paresis. A certain minimum dose of local anesthetic is necessary to provide either surgical anesthesia or analgesia. For example, the superior trunk block and costoclavicular block have demonstrated efficacy using 15 mL of bupivacaine 0.5% and 20 mL of levobupivacaine 0.5%, respectively [7,31]. By limiting the volume of local anesthetic, the risk of proximal spread of local anesthetic to the phrenic nerve and resulting diaphragmatic paresis is reduced. 

Ultimately, the importance of providing effective analgesia or even surgical anesthesia with a nerve block needs to be weighed against the risk of potential phrenic nerve paresis. Fortunately, there are an increasing number of regional techniques with significantly lower risk of respiratory effects. With newer techniques and greater clinician experience, regional anesthesia may offer important benefits for patients undergoing shoulder surgery while minimizing the downsides that have typically been associated with these techniques.

## Figures and Tables

**Table 1:** Novel regional anesthesia techniques for shoulder surgery.

Type	Procedures Studied in RCT	Full Surgical Anesthesia?	Efficacy vs. ISB *	Incidence of HDP *
STB	Arthroscopic shoulder surgery	Yes	Similar	4.8–26.7%
SSNB-ANB	Arthroscopic shoulder surgery	No	Inferior	No RCT data
ICB-SSNB	Arthroscopic shoulder surgery	Yes	Similar; slightly higher morphine consumption at 24 h	0–3%
CCB	Arthroscopic shoulder surgery	Yes	Similar	0–7.6%

STB = Superior Trunk Block, SSNB-ANB = Combined axillary and suprascapular nerve block, ICB-SSNB = Combined infraclavicular and suprascapular nerve block, CCB = Costco-clavicular block, * based on RCT data. RCT = Randomized controlled trial; ISB = Interscalene nerve block; HDP = Hemi-diaphragmatic paralysis.

## Data Availability

This study did not create new data.
